# How to get better TAD? Relationship between anteversion angle of nail and position of femoral neck guide pin during nailing of intertrochanteric fractures

**DOI:** 10.1186/s12891-020-03518-5

**Published:** 2020-08-01

**Authors:** Zhe Wang, Yadong Liu, Shenglong Li, Xiuhui Wang, Changjian Liu, Xin Tang

**Affiliations:** 1 Department of Orthopedics, Zhongshan Hospital, Fudan University, Shanghai, 200032 China; 2 Department of Orthopedic Trauma, the First Affiliated Hospital of Dalian Medical University, Dalian, 116011 Liaoning Province China; 3 Department of Bone and Soft Tissue Tumor Surgery, Cancer Hospital of China Medical University Liaoning Cancer Hospital & Institute, Shenyang, 110042 Liaoning Province China; 4 Department of Orthopedics, Shanghai University of Medicine & Health Sciences Affiliated to Zhoupu Hospital, Shanghai, 201318 China

**Keywords:** Intertrochanteric fracture, 3D modeling, Internal-fixation, Guide pin, Tip–apex distance, Pin-shaft angle

## Abstract

**Background:**

To demonstrate the correlation between guide pin-shaft angle (PSA) at the anteroposterior film and anteversion angle of guide pin at the lateral film and investigate whether excellent tip–apex distance (TAD) can be obtained by changing the entry point via axial rotation of the main intramedullary nail.

**Methods:**

Fifty patients with intertrochanteric femoral fractures (IFFs) undergoing internal fixation with intramedullary nails under 2D fluoroscopy were retrospectively enrolled. Both of the PSA at the anteroposterior film and anteversion angle at the lateral film before and after adjustment of the guide pin were collected. Pearson correlation analysis was performed to investigate their correlation. Intraoperative and postoperative outcomes were recorded. Furthermore, the software of Mimics 10.0 and Pro/E were used to establish the 3D models of the proximal femur and main intramedullary nail/guide pin, respectively. Surgery was simulated on the Pro/E software platform and solid geometry analysis was conducted to calculate the correlation between the PSA and the anteversion angle.

**Results:**

Pearson correlation analysis indicated there was a positive correlation between PSA and anteversion angle, with the correlation coefficient of 0.902 (*p* < 0.01). By altering the PSA and anteversion angle, TAD was adjusted to be less than 25 mm in all patients. The mean operative time, fluoroscopy time and length of hospital stay were 65.82 ± 11.16 min, 2.03 ± 0.79 min and 6.66 ± 2.49 d. Thirty-one patients received blood transfusions (3.55 ± 1.95 U). Fracture reduction was considered to be good or acceptable in all patients. Complications occurred only in 6 patients (12.00%). At a 3-month follow-up, the mean Timed Up and Go was 31.54 ± 20.95 s and Harris Hip Score was 72.88 ± 8.79. The 3D surgery model also showed when the main intramedullary nail was externally rotated or internally rotated of 20° at the standard location, the PSA of guide pin at the anteroposterior position and anteversion angle of the guide pin at the lateral position were simultaneously increased or decreased.

**Conclusion:**

Our findings suggest altering the PSA and anteversion angle may be beneficial for obtaining excellent TAD and achieving superior outcomes.

## Background

Intertrochanteric femoral fractures (IFFs) are relatively common clinical injuries in elder people (especially aged over 65 years), accounting for approximately 50% of all hip fractures [1]. With the improvement of living standards, the increase of serious traffic accidents and the extension of human life, the incidence of IFFs is reported to be markedly rising, particularly in Asia [2]. It is estimated that the total number of hip fractures may annually exceed 0.5 million in 2030 and 1 million in 2050 in Asia [3]. Thus, how to manage IFFs has been a hot issue for orthopedic surgeons.

Currently, surgical intervention is recommended as the preferred treatment for IFFs except a few patients who had a very poor general condition and can’t tolerate anesthesia and surgery. Generally, intramedullary [i.e. proximal femoral nail anti-rotation (PFNA), proximal femoral nail (InterTAN), Gamma nail] and extramedullary (i.e. dynamic hip screw, dynamic condylar screw) fixations are two primary options for surgical treatment of such fractures. Compared with extramedullary implants, less blood loss, mechanical complications and better functional scores can be achieved after intramedullary fixations [4, 5]. Further comparisons between different intramedullary fixators indicate PFNA and InterTAN may be superior to Gamma nail to cause shorter operative time, length of hospital stay and less blood loss [6, 7], while a similar effect may be present between PFNA and InterTAN [8]. Therefore, intramedullary nail fixations with PFNA and InterTAN are the most widely used approaches for IFFs.

It has been a consensus that measuring the tip–apex distance (TAD) on anteroposterior and lateral radiographs is a valuable tool to assist accurate placement of screws or nails for fixation of IFFs. The TAD should be less than 25 or 27 mm in order to reduce the complications of the cut-out and nail breakage, fracture non-union and poor functional outcomes [9–13]. However, intraoperative determination of TAD is difficult and can be hampered by the image quality, body habitus and image projection [14, 15]. The repetitive adjustment of the main intramedullary nail and guide pin under the C-arm machine or a wide range of stripping and exposure may further increase the operative and fluoroscopy time and cause more complications and instability, especially for the surgeons who are lack of experience [16–19]. Hereby, how to quickly obtain this excellent TAD remains a challenging problem. In our clinical experience, we occasionally found an interesting phenomenon: there is a correlation between pin-shaft angle (PSA, ∠α, defined as the angle between the axis of the main femoral intramedullary nail and the axis of the screw guide pin of the femoral neck at the anteroposterior position) and anteversion angle (∠β, defined as the angle between the main axis of the femoral intramedullary nail and the axis of the screw guide pin of the femoral neck at the lateral position) when the main femoral intramedullary nail was rotated along the axis during the operation to adjust the location of femoral neck screw guide pin. With the main intramedullary nail keeping in constant depth, when the anteversion angle was increased, there was also an increase in the PSA, which led to an elevated place of the guide pin in the head neck and caused the change in TAD. This provided an idea to regulate TAD by altering the PSA and anteversion angle.

In this study, we aimed to further quantize the correlation between the PSA and the anteversion angle using the imaging data of case series and assess whether altering the PSA and anteversion angle may be beneficial for obtaining excellent TAD and achieving superior intraoperative and postoperative outcomes. Furthermore, three-dimensional (3D) operation and mathematical simulations [20] were also used to establish a personalized distribution map of the femoral neck guide pin to guide the location adjustment for the femoral neck guide pin.

## Methods

### Clinical trials

Patients with IFFs who underwent closed reduction and internal fixation with femoral intramedullary nails under two-dimensional (2D) fluoroscopy in the Zhongshan Hospital, Fudan University, the First Affiliated Hospital of Dalian Medical University and Shanghai Pudong New District Zhoupu Hospital between March 2010 and October 2015, were retrospectively enrolled. Inclusion criteria were: (1) age > 65 years; (2) the time interval from the injury to admission < 24 h; (3) intraoperative fracture end was well reset and the anatomical repositioning was generally achieved; (4) the position of the guide pin in the femoral neck was adjusted by changing the entry point via axial rotation of the main intramedullary nail; (5) the imaging data were completed; and (6) unilateral closed fracture. Exclusion criteria included: (1) concurrent femoral fractures at other parts of the same side; (2) long-term wheelchair use before injury; (3) pathological IFFs; (4) old IFFs; (5) without fixed address or some subjective reasons not want to participate in the trials; and (6) incomplete follow-up information. This study was approved by the ethics committee of our institution.

**Fig. 1:**
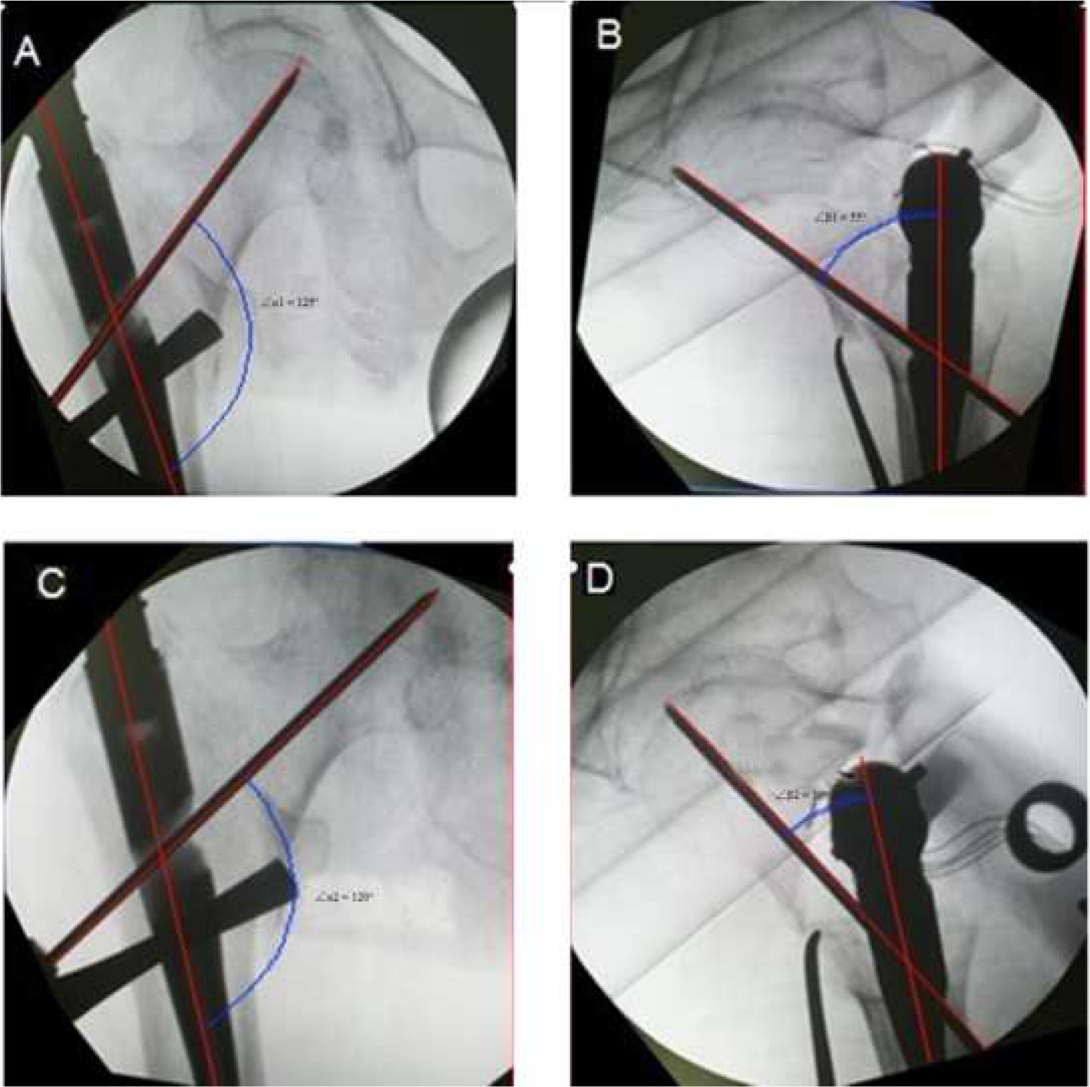
The guide pin-shaft angle and anteversion before (**a**,∠α1; **c**, ∠β1) and after (**b**, ∠α2; **d**, ∠β2) the adjustment of the guide pin measured for one included patient

All surgery in three centers was performed by the same senior surgeon under general, continuous epidural or nerve block anesthesia. The main intramedullary nail was placed into the proper location in the femoral shaft according to the standard surgical procedures: First, the guide pin was placed into the femoral neck, and non-ideal location of the guide pin was seen under fluoroscopy at the anteroposterior and lateral positions during the operation; Then, the main nail was rotated along its longitudinal axis to adjust the entry point of guide pin followed by replacement of the guide pin and observation under fluoroscopy. The length of the main intramedullary nail and mode of distal locking were determined according to the patients’ condition. The location of the femoral neck guide pin was adjusted by axially rotating the main femoral intramedullary nail to alter the entry point and ensure the TAD not exceeding 25 mm [9–13]. The TAD was measured by three individual observers through C-arm x-ray intra-operatively. The rotation of the nail will not stop until guide pin is on an ideal position. So, the nail should be rotated slowly and steadily with the assistance of intra-operative c-arm X-ray to decide the rotation of the nail. Meanwhile, the rotation angle of the nail was recorded based on the location change in pre- and post- rotation of T-shaped wrench. PSA and anteversion angle before (∠α1, ∠β1) and after (∠α2, ∠β2) the adjustment of the guide pin was respectively measured for all the included patients using professional drawing software on X-ray (Fig. 1). In order to reach a good reduction for the proximal femur, the intact opposite proximal femur was also x-rayed preoperatively to provide a reference for reduction procedure during the surgery. The angle of the nail was chosen for fracture fixation based on neck-shaft angle from the opposite femur.

At 24 h after operation, patients were encouraged to walk with the aid of double crutches or walker. Partial weight-bearing was also allowed on the affected limb. Rivaroxaban was orally administered for 7 weeks to prevent thrombogenesis. The operative time (min), overall fluoroscopy time (min), blood loss during surgery, amount of transfused blood (U), length of hospital stay (d) and postoperative complications were recorded. The radiographs of the hip joints (anteroposterior position, bilateral; lateral position, the affected) were made to evaluate the fracture reduction at 24–48 h after operation. The quality of the fracture reduction was graded as poor (> 10^°^ varus/valgus), acceptable (5^°^ – 10^°^ varus/valgus) or good (< 5^°^ varus/valgus) [21]. Functional outcome was assessed postoperatively, at 6-week and 3-month follow-up on the basis of the Timed Up and Go (TUG) test [22] and the Harris hip score (HHS) [23].

Data were expressed as mean ± standard deviation or number (%). Pearson correlation analysis was performed to investigate the correlation between the PSA at the anteroposterior position and anteversion angle of the guide pin at the lateral position. The level of statistical significance was set at a two-sided *p*-value < 0.05.

### Simulation analysis

**Fig. 2:**
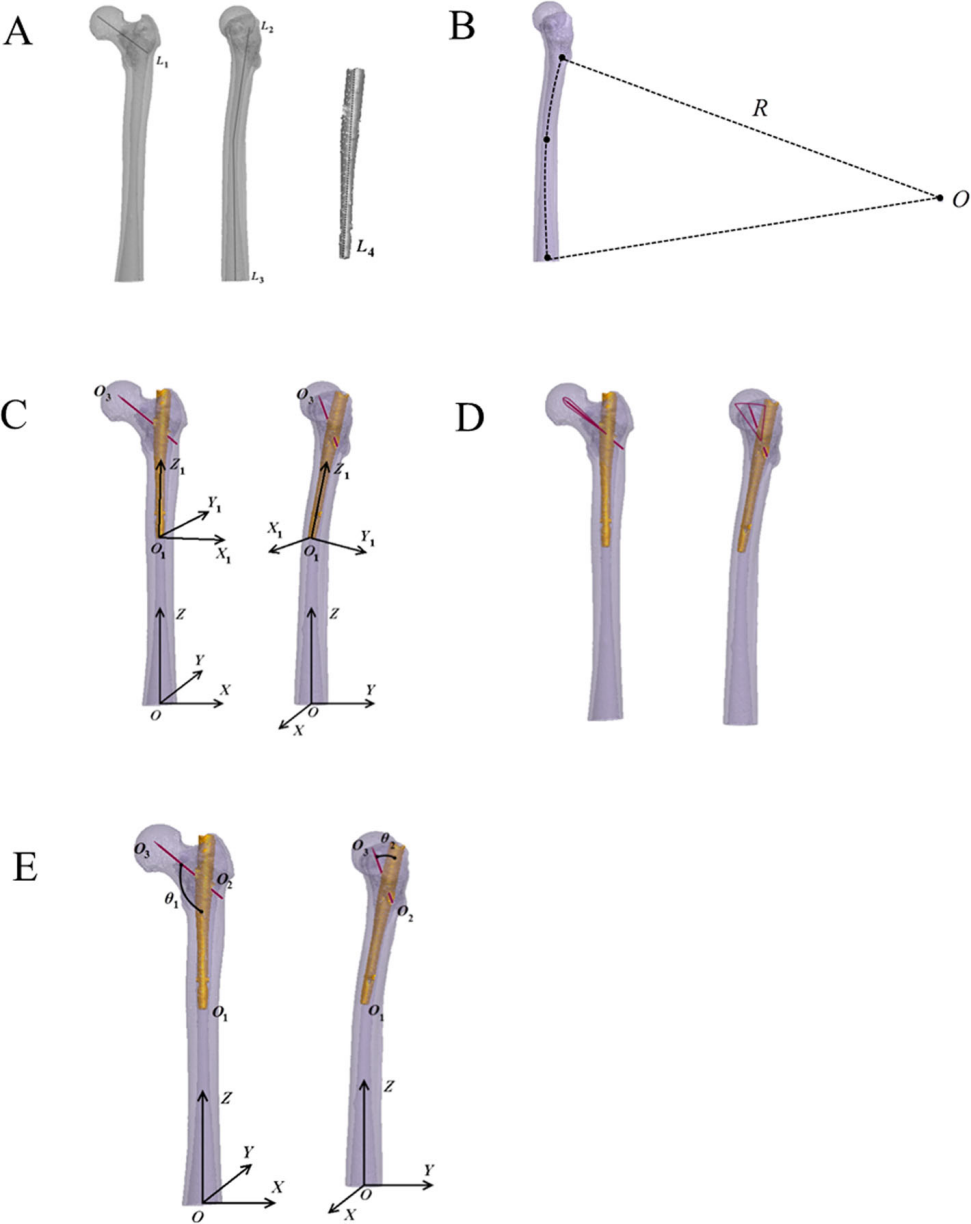
Simulation operation and mathematical analysis. **a**, femoral and intramedullary nail model; **b**, measurement on curvature radius of femur; **c**, fluoroscopycoordinate system O-XYZ, guide pin trajectory co-ordinate system O1-X1Y1Z1; **d**, The plane of the femoral neck guide pin is within the scope of the red line; **e**, calculation on PSA of and anteversion of guide pin

Among the included patients, one patient was randomly selected to extract the raw computerized tomography (CT) data and establish the femoral and intramedullary nail geometric models. Data requirements included: (1) the patient underwent postoperative 64 slice spiral CT scan (GE company, USA), with the scan scope from the hip to the lower 1/3 of the femur; (2) the slice gap of each scan was 1 mm; (3) the density of pixel matrix was 512 × 512; and (4) 2 bytes were assigned for each pixel. The obtained CT images were stored in Dicom format. Patient’s data in Dicom format were imported into Mimics 10.01 (Materialize company, Belgium). Through image positioning, threshold setting, dynamic area growth and hole filling, the redundant data were removed and the 3D visualization model of the femur (Fig. 2a) was rebuilt. According to PFNA intramedullary nailing data provided by SYNTHES (Switzerland), the intramedullary nail and guide pin models (Fig. 2a) were established using Pro/E 3.0 software. Models were exported in a .lis format. The femoral shaft was bent to the anterolateral direction. Mimics 10.01 was used to draw the axis of the femoral marrow cavity, and the physical radian of the marrow cavity axis from the site of 2 cm under femoral intertrochanter to lower 1/3 of the femur was measured and presented as curvature radius (R) (Fig. 2b). Detail processes of operation simulation and mathematical analysis (Fig. 2c-e) were described in Supplementary file 1.

## Results

### Clinical observation

Fifty patients were included in this study (Table 1; Supplementary file 2), where 30 (60%) cases were male and 20 (40.0%) cases were female. Their age ranged from 60 to 85 years old, with an average of 76.04 ± 6.28 years old. All the patients suffered from IFFs with 28 (56.00%) occurred on the left side and 22 (44.00%) on the right side. Unstable trochanteric fractures were classified as AO – 31A2 in 46 (92.00%) cases and 31A3 in 4 (8.00%) cases. All of the fractures were fresh closed fractures and were treated with closed reduction and internal fixation with femoral intramedullary nails.

**Table 1:** Demographics of 50 patients

	Patients
Gender (female)	
Age (year)	76.04 ± 6.28
Side (left)	28 (56.00%)
Injury mechanism	
Traffic injury	8 (16.00%)
Falling injury	42 (34.00%)
AO type	
31A2	46 (92.00%)
31A3	4 (8.00%)
ASA	
1	1 (2.00%)
2	23 (46.00%)
3	25 (50.00%)
4	1 (2.00%)

**Fig. 3:**
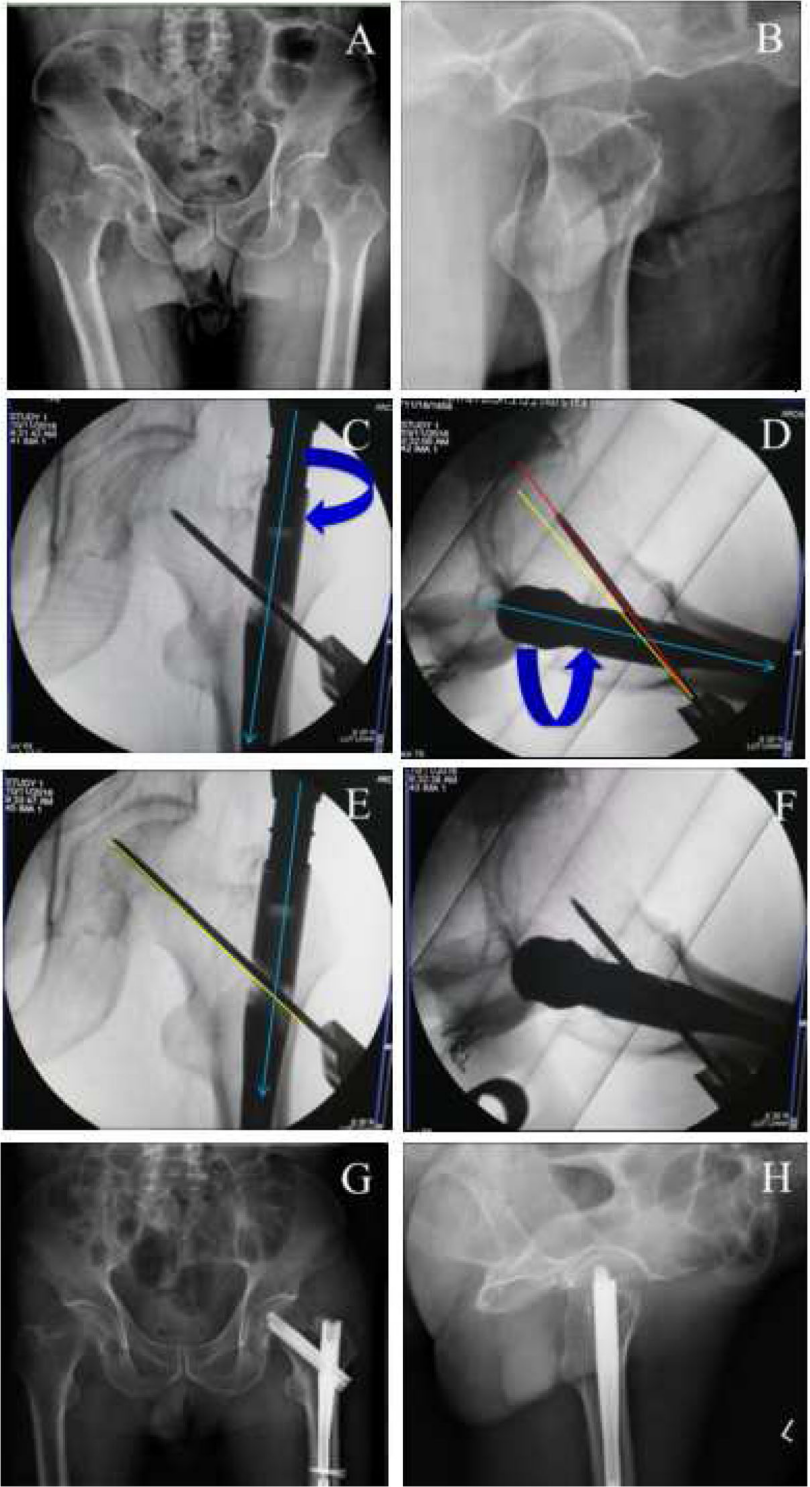
A typical case to regulate the PSA and anteversion angle to obtain ideal TAD. **a**-**b**, preoperative anteroposterior (**a**) and lateral (**b**) X-ray to diagnose intertaochanteric fractures; **c**-**f**, the adjustment of the guide pin. When position of neck guide pin is higher (the PSA is larger, **c**) and anteversion angle of nail is more than normal (**d**), we need to internally rotate the nail to make anteversion angle smaller (**f**) (at the same time, the PSA will become less in AP view, **e**) to get better TAD; **g**-**h**, postoperative anteroposterior (**g**) and lateral (**h**) X-ray to confirm the fracture reduction. Green line, nail position; red line, current position; yellow, ideal position

**Fig. 4:**
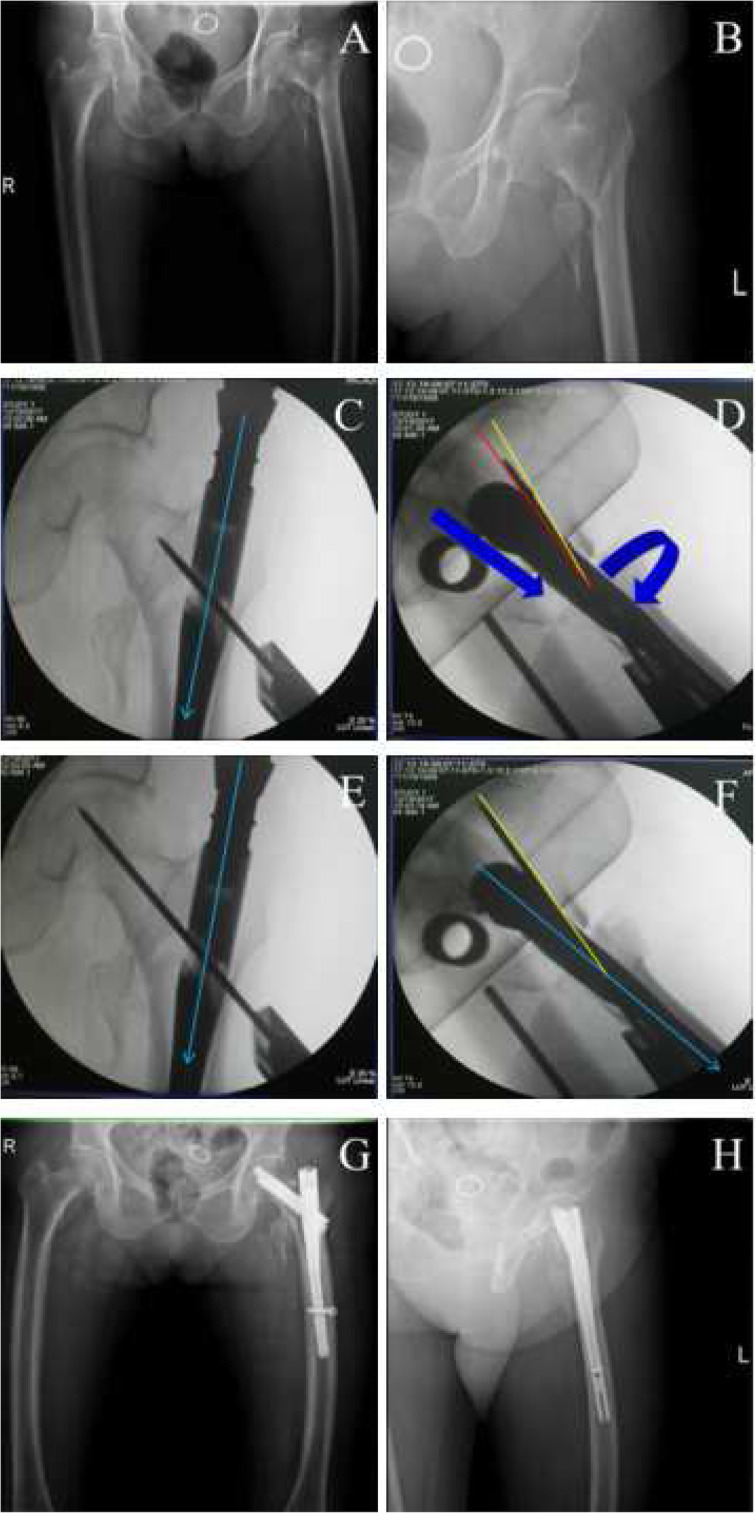
A typical case to regulate the PSA and anteversion angle to obtain ideal TAD. **a**-**b**, preoperative anteroposterior (**a**) and lateral (**b**) X-ray to diagnose intertaochanteric fractures; **c**-**f**, the adjustment of the guide pin. When position of neck guide pin is higher (the PSA is larger, **c**) than normal and anteversion angle is less than normal (**d**), we need to deepen the nail and externally rotate it to make antevertion angle bigger (**f**) (at the same time, the PSA will increase in AP view, **e**) to get better TAD; **g**-**h**, postoperative anteroposterior (**g**) and lateral (**h**) X-ray to confirm the fracture reduction. Green line, nail position; red line, current position; yellow, ideal position

**Fig. 5:**
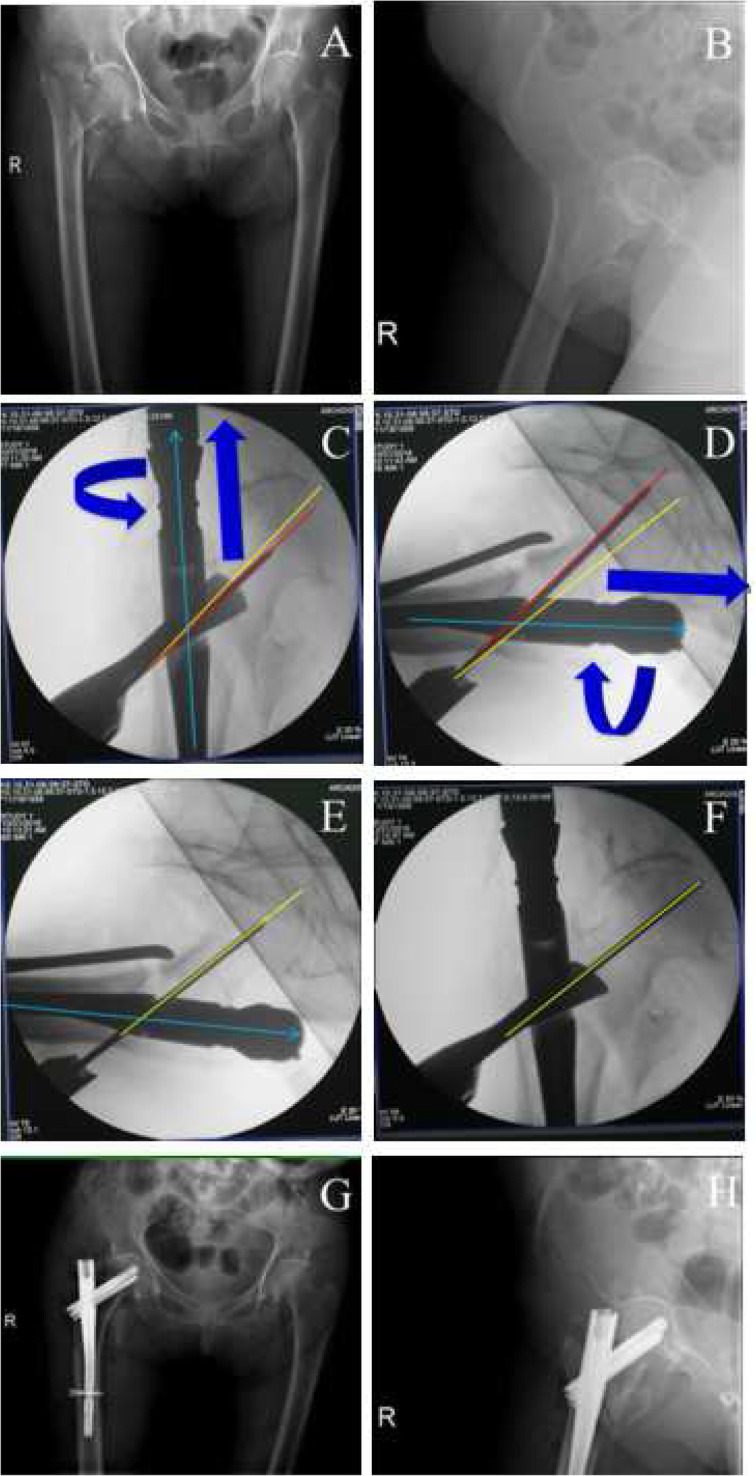
A typical case to regulate the PSA and anteversion angle to obtain ideal TAD. **a**-**b**, preoperative anteroposterior (**a**) and lateral (**b**) X-ray to diagnose intertaochanteric fractures; **c**-**f**, the adjustment of the guide pin. When position of neck guide pin is lower (the PSA is less, **c**) than normal and anteversion angle of the nail is more than normal (**d**), we need to partially extract the nail and internally rotate it to make anteversion angle less (**f**) (at the same time, the PSA will decrease in AP view, **e**) to get better TAD; **g**-**h**, postoperative anteroposterior (**g**) and lateral (**h**) X-ray to confirm the fracture reduction. Green line, nail position; red line, current position; yellow, ideal position

**Fig. 6:**
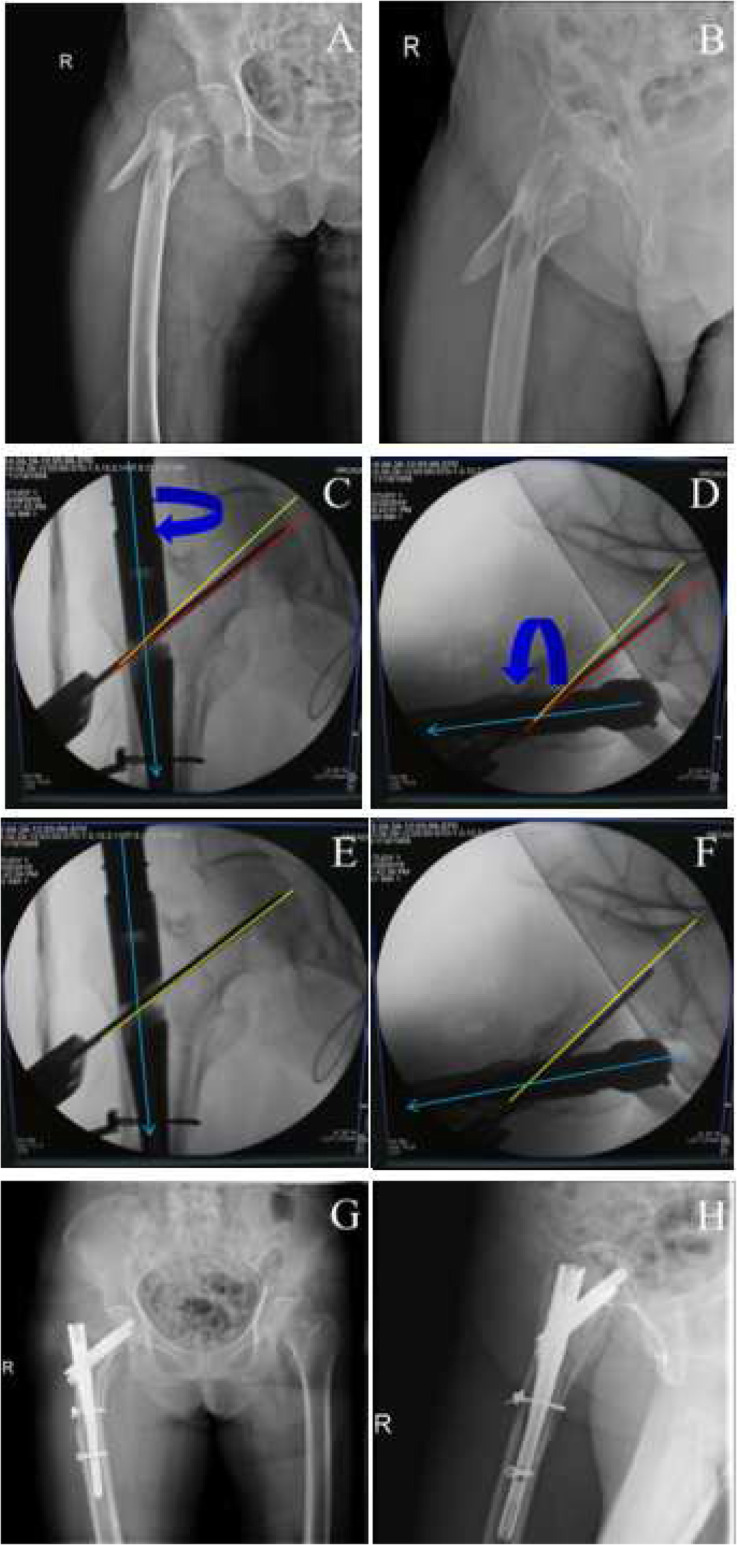
A typical case to regulate the PSA and anteversion angle to obtain ideal TAD. **a**-**b**, preoperative anteroposterior (**a**) and lateral (**b**) X-ray to diagnose intertaochanteric fractures; **c**-**f**, the adjustment of the guide pin. When position of neck guide pin is lower (the PSA is larger, **c**) and anteversion angle of nail is less than normal (**d**), at first, we need only to externally rotate the nail to make antevertion angle bigger (**f**) (at the same time, the PSA will become larger in AP view, **e**) to get better TAD; **g**-**h**, postoperative anteroposterior (**g**) and lateral (**h**) X-ray to confirm the fracture reduction. Green line, nail position; red line, current position; yellow, ideal position

PSA (∠α) and anteversion angle (∠β) of the guide pin on intraoperative imaging were collected from these 50 clinical cases (Table 2) and then, Pearson correlation analysis was performed. In line with our occasionally seen results, there was indeed a significantly positive correlation between the PSA and the anteversion angle, with the correlation coefficient of 0.902 (*p* < 0.01). By altering the PSA and the anteversion angle (Figs. 3, 4, 5 and 6), TAD was adjusted to be less than 25 mm in all patients (20.52 ± 2.80 mm). Fracture reduction (Figs. 3, 4, 5 and 6) was considered good or acceptable in all 50 patients on post-operative radiographs (Table 2).

**Table 2:** Operative records

	Data
Operation time (min)	65.82 ± 11.16
Blood transfusion (U)	3.55 ± 1.95
Fluoroscopy time (s)	2.03 ± 0.79
Length of hospital stay (d)	6.66 ± 2.49
Fracture reduction	
Good	47 (94.00%)
Acceptable	3 (6.00%)
Poor	0
∠α1	131.63 ± 5.27
∠β1	26.69 ± 7.36
∠α2	130.30 ± 3.72
∠β2	21.55 ± 3.84
TAD (mm)	20.52 ± 2.80

*TAD* Tip–apex distance; The pin shaft angle and anteversion before (∠α1, ∠β1) and after (∠α2, ∠β2) the adjustment of the guide pin

The mean operative time, fluoroscopy time and length of hospital stay were 65.82 ± 11.16 min, 2.03 ± 0.79 min and 6.66 ± 2.49 d. Thirty-one patients received postoperative blood transfusions, with the amount of transfused blood of 3.55 ± 1.95 U (Table 2). Complication occurred in 6 patients (10.90%), including urinary tract infection in 2 (4.00%), bronchopneumonia in 1 (2.00%), hypoglycemia in 1 (2.00%), delirium in 2 (4.00%), uroschesis in 1 (2.00%), lower limb vein thrombosis in 1 and vulvar ulcerations in 2 (4.00%) (Table 3). The mean TUG and HHS were 123.60 ± 12.78 s and 52.14 ± 13.78 postoperatively, 47.52 ± 27.22 s and 63.04 ± 10.72 at 6-week, 31.54 ± 20.95 s and 72.88 ± 8.79 at 3-month follow-up (Table 4).

**Table 3:** Postoperative complications

	Data
Urinary tract infection	2 (4.00%)
Bronchopneumonia	1 (2.00%)
Hypoglycemia	1 (2.00%)
Delirium	2 (4.00%)
Uroschesis	1 (2.00%)
Lower limb vein thrombosis	1 (2.00%)
Vulvar ulcerations	2 (4.00%)

**Table 4:** Function outcomes

	Data
TUG test (s)	
Postoperative	123.60 ± 12.78
6-week	47.52 ± 27.22
3-month	31.54 ± 20.95
Harris Hip Score	
Postoperative	52.14 ± 13.78
6-week	63.04 ± 10.72
3-month	72.88 ± 8.79

*TUG* Timed Up and Go

### Model validation

The curvature radius of the medullary cavity was 90.02 cm. The virtual surgery was realistic with good 3D visual effects. The design of virtual surgery was in accordance with the actual intraoperative situation. The axis of the main intramedullary nail coincided with the axis of the upper femur. The depth of insertion was proper. The guide pin of tension screw was located at the axis of the femoral neck, and the TAD was 22 mm.

**Fig. 7:**
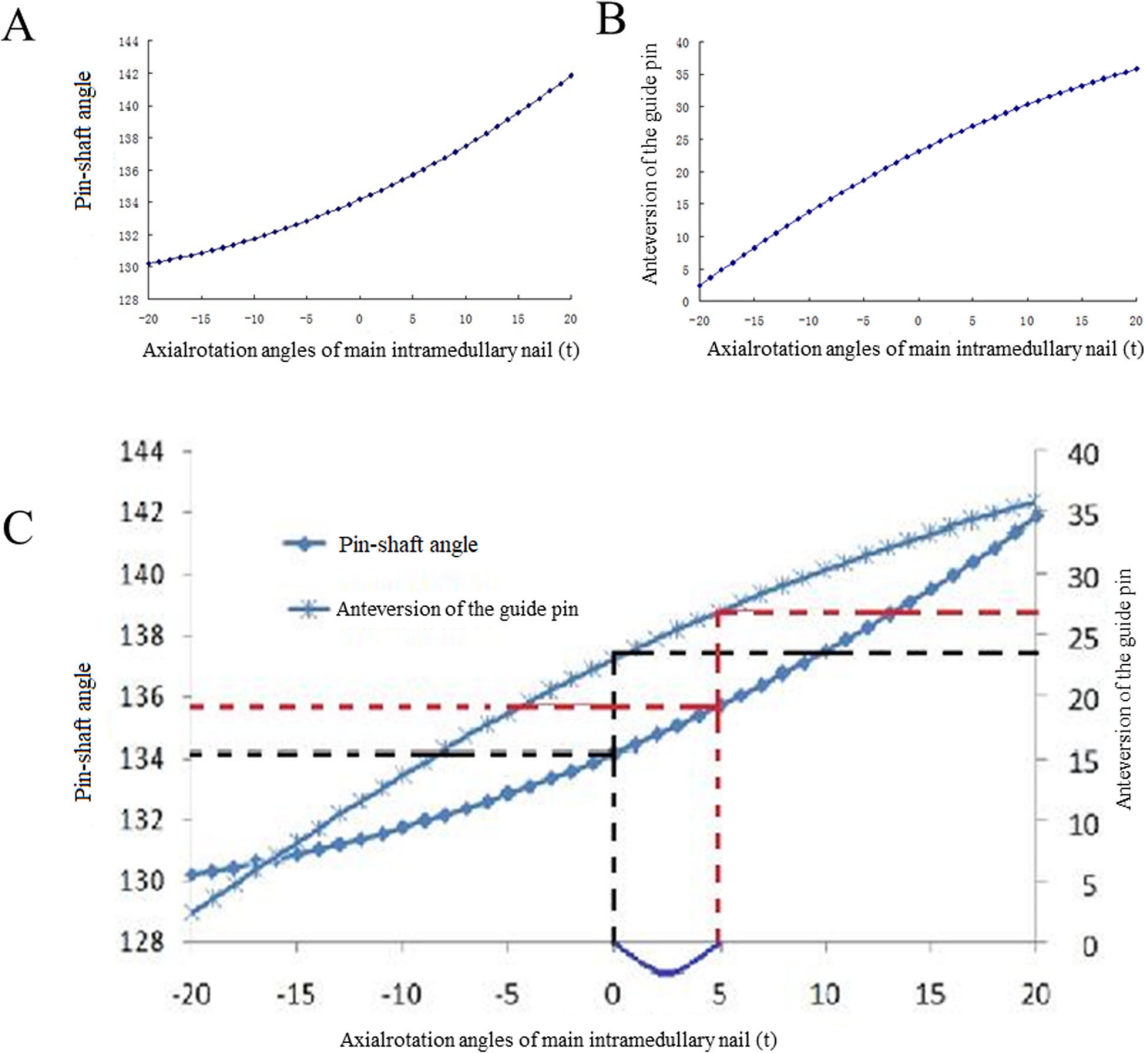
Personalized position map of femoral neck guide pin. **a**, the relationship between neck shaft angle and anteversion angle of the guide pin when axial rotation angle of main intramedullary nail changed; **b**, application of personalized distribution map of femoral neck guide pin. The coordinates of the black line was corresponding to the ideal location of femoral neck guide line in preoperative planning. PSA of guide pin is 135.7°, while anteversion of guide pin is 24°. The coordinates of the red line was corresponding to the first position of femoral neck guide line in operation; based on the PSA and anteversion angle of guide pin measured in the operation, the corresponding positions in the figure are found; the abscissa and the rotation angle of the main intramedullary nail are found; compared with the abscissa of red and black line, the method to adjust the guide pin location can be obtained

When the main intramedullary nail was externally and internally rotated of 20° at the standard location, the trajectory of the endpoint of the guide pin was curved. Under the fluoroscopy coordinate system O-XYZ, with the axial rotation angle of the main intramedullary nail (t) as the variable, the changes of the PSA at the anteroposterior position and anteversion angle of the guide pin at the lateral position were observed through calculation (Fig. 7). The PSA at the anteroposterior position and anteversion angle of the guide pin at the lateral position were both increased along with the increase in the t value when the main intramedullary nail was externally rotated (Fig. 7a); while they were decreased along with the decrease in the t value when the main intramedullary nail was internally rotated (Fig. 7b).

According to the correlation between the PSA and the anteversion angle of the guide pin, a personalized position map of the femoral neck guide pin was designed and developed (Fig. 7c). The abscissa was the axial rotation angle of the main intramedullary nail, while the ordinate values were respectively the PSA of guide pin at the anteroposterior position and the anteversion angle of the guide pin at the lateral position. t = 0 was defined as the ideal position of the guide pin in the femoral neck. Based on the figure, the PSA of guide pin at the anteroposterior position was 134°, while anteversion angle of the guide pin at the lateral position was 25° at the corresponding site.

## Discussion

In the present study, we used the CT data of clinical IEF patient series and 3D mathematical model to demonstrate there was a positive correlation between the PSA and the anteversion angle. By synergistically altering the PSA and theanteversion angle, TAD can be easily adjusted to be less than 25 mm (20.52 ± 2.80 mm) which is recommended by several scholars during the fixation of IEFs in order to prevent complications [24, 25]. The accessibility and perfection of TAD adjustment in our study smoothed the surgery (shorter operative time: 65.82 ± 11.16 min vs 79.50 ± 21.12 min [21, 26, 27] and fluoroscopy time: 2.03 ± 0.79 min vs 2.9 ± 0.16 min) [7]), improved the treatment effects (higher HHS: range, 57–89 vs 36–97 [28]) and decreased the complications (rate: 12.00%, 6/50 vs 57.44%, 108/188 [21]; cut-out: 0% vs 4% [29] or 2.7% [30]) compared with previous studies where TAD was higher than 25 mm, even modified 30 mm [31] in some cases [28–30].

There was evidence to show the potential correlation between the neck-shaft angle and TAD in IEF patients. For example, Boukebous et al. calculated the neck-shaft angle gap between the fractured and the healthy sides and found the average TAD can reach 27 mm in patients with a 7% eck-shaft angle gap rate [32]. Walton et al. demonstrated the number of patients with TAD greater than 25 mm was more in the group with the neck-shaft angle < 125° compared to those having the neck-shaft angle > 125° (27.8% vs 12.6%) [33]. Also, a positive association was observed between the neck-shaft angle and hip position [34, 35]. However, the role of anteversion angle, as well as its corresponding changes induced by rotation of the nail and how the changes of PSA and anteversion angle to regulate the TAD in IFFs has not been further investigated. Our study was, for the first time, to demonstrate their synergetic relationship and may provide a novel method to quickly regulate TAD in the clinic via the preoperative establishment of the personalized distribution map of femoral neck guide pin (Fig. 7) [36]. But, one thing should be paid attention: the curvature of anterolateral femoral bowing is usually increased in the elderly, which may lead to the mismatching between the cephalomedullary nail and the femur and cause cortical impingement and tail protrusion [37–40]. For these patients, we performed the following procedures to prevent these complications: 1) using the long main nail; 2) the nail entry point should be selected anterior, but not posterior to the greater trochanter; and 3) when the excellent PSA of the guide pin was achieved by rotation of the main nail, the traction force can be slightly elevated. After the TAD was adjusted, the traction force can be restored to the initial level.

However, there were some limitations to this study. First, the application of a personalized distribution map of the femoral neck guide pin must meet the following conditions: 1) The femur at the diseased side has achieved anatomical repositioning. In these patients, the slight rotation of the main mail to change the PSA and the anteversion angle may not influence its reduction stability and affect the position of blade in femoral head (Figs. 4, 5 and 6); 2) Fluoroscopy plane in preoperative simulation surgery should be in accordance with the actual fluoroscopy during the operation; 3) It fits for adjusting the position of the guide pin in the femoral neck by axial rotation of the main intramedullary nail, but not fit for adjusting the depth of the main intramedullary nail; and 4) The anatomical and fluoroscopy position of the patient must not be changed during fluoroscopy. Second, the experiment design itself also has some limitations. 1) The 3D femoral model in this paper was established based on the patients with preoperative CT scan data and the projection plane may deviate to the actual projection; 2) Engineering software commonly used in digital orthopedics is not designed aiming at orthopedics system, but reference to some mechanical engineering software. When dealing with complex skeleton model, this software may develop some error for the measurement data; 3) Preoperative design and simulation work needs time and improved preoperative imaging tests, which are difficult to apply to patients needing emergency surgery; and 4) the patients were retrospectively enrolled without control and sample size was relatively small and follow up was short, which may result in the underestimation of the complication rate and prognosis. Thus, a prospective study demonstrating the technique intraoperatively will be necessary for the future.

## Conclusion

Through analysis of clinical cases and research on the 3D model, our findings suggest there is a positive correlation between anteversion of the femoral neck guide pin at the lateral position and the PSA of guide pin at the anteroposterior position. When the main intramedullary nail was rotated along the axis intraoperatively to change the position of the guide pin, as the anteversion of the guide pin was increased, the PSA was also increased. Otherwise, as the anteversion of the guide pin was decreased, the PSA was also decreased. Based on this principle (Table 5), the location of the femoral neck guide pin can be adjusted to get better TAD simply and accurately during operation, which ensures the excellent intraoperative and postoperative outcomes.

**Table 5:** Tips for the adjustment of the guide pin

Guide pin position (AP)	Anteversion of guide pin (Lateral)	Adjustment protocol	Projection PSA (AP)
Superior (to ideal position)	Greater	1. Decrease the antevertion of nail by intorsion of the nail	Decrease
Superior (to ideal position)	Less	1. Deepen the nail 2. Increase the anteversion by extorsion of the nail	Increase
Inferior (to ideal position)	Greater	1. Pull out the nail 2. Decrease the anteversion of guide pin by the intorsion of the nail	Decrease
Inferior (to ideal position)	Less	1. Increase the anteversion of guide pin by the extorsion of the nail	Increase

*PSA* Pin shaft angle, *AP* Anteroposterior

## Supplementary information

**Additional file 1.** Processes of operation simulation and mathematical analysis.

**Additional file 2.** Patients’ data.

## Data Availability

All data generated or analyzed during this study are included in this published article.

## Abbreviations

IFFs: Intertrochanteric femoral fractures
PFNA: Proximal femoral nail anti-rotation
InterTAN: Proximal femoral nail
TAD: Tip–apex distance
PSA: Pin-shaft angle
3D: Three-dimensional
2D: Two-dimensional
TUG: Timed Up and Go
HHS: Harris hip score
CT: Computerized tomography
ASA: American Society of Anesthesiologists
