# Supplementary material for: How to get better TAD? Relationship between anteversion angle of nail and position of femoral neck guide pin during nailing of intertrochanteric fractures
Source: BMC Musculoskelet Disord. 2020 Aug 1;21:512. doi: 10.1186/s12891-020-03518-5 (PMC7395984; doi:10.1186/s12891-020-03518-5)
Supplement: Supplementary file 1 — Additional file 1. Processes of operation simulation and mathematical analysis. [file 12891_2020_3518_MOESM1_ESM.docx]

**Methods**

Operation simulation was performed as following:

1) Data files of femur, main intramedullary nail and guide pin in the .lis format were imported into software Pro/E 3.0, and respectively saved as Pro/E components;

2) Axes of the femoral neck (*L1*), proximal femoral shaft (*L2*) and distal femoral shaft (*L3*) were found and established through measurement in the model of femoral component, while axis of the main nail (*L4*) was established in model of main intramedullary nail. In model of guide pin, axis of guide pin (*L5*) was also found and established;

3) New assembly was established in the software Pro/E, and the model of femoral component was imported and fixed on the acquiescent default location; component model of main intramedullary nail was inserted along the axis of proximal femoral shaft (*L2*), to recombine *L2* and *L4* and adjust the main nail to the appropriate depth. The positions of main intramedullary nail and guide pin of femoral neck were fixed with the angle of 130°. Guide pin of femoral neck screw was placed; the guide pin and main intramedullary nail component were set as a whole, called intramedullary nail composite. With axes *L1* and *L5* as well as *L2* and *L4* as the reference, the position of intramedullary nail composite in the femoral model was adjusted until it met the clinical standards.

Mathematical analysis was performed according to the following steps:

1) With the lower endpoint of the axis of distal femoral shaft *(L3)* as the origin of coordinates (O) and the axis of distal femoral shaft *(L_3_)* as the Z axis, the plane through Z axis and parallel with the axis of femoral neck *(L_1_)* was defined as plane XOZ. The fluoroscopy-coordinate system O-XYZ was established according to the right-hand rule. Anteroposterior fluoroscopy was defined as positively along the Y axis (XOZ plane projection), while lateral position fluoroscopy was defined as reversely along the X axis (YOZ plane projection);

2) With the lower endpoint of the axis of main intramedullary nail as the origin of coordinates (O_1_), and the axis of main intramedullary nail *(L_4_)* as the Z1 axis, the plane of the axis of the main intramedullary nail and the axis of guide pin was defined as plane X_1_O_1_Z_1_, and guide pin trajectory coordinate system O_1_-X_1_Y_1_Z_1_ was established according to the right-hand rule (Figure 2C);

3) After assembly, the main intramedullary nail and the femoral neck guide pin were considered as a whole. Through axial rotation of the main intramedullary nail, the position of guide pin in the femoral neck was changed. When the main nail was rotated along its axis, the trajectory of the endpoint of the guide pin was a period of circular arc. The coordinate of the endpoint of the guide pin can be determined based on the relative positions of femur and intramedullary nail with the sizes of femur and intramedullary nail. Then, the changes of the angle between axis of main intramedullary nail and axis of guide pin under fluoroscopy at the anteroposterior and the lateral position can be determined, namely the changes of PSA and anteversion angle (Figure 2D);

4) Fluoroscopy coordinate system O - XYZ was the fixed coordinate system. Based on the measured data, the origin of coordinates O1 of the guide pin trajectory coordinate system O1-X_1_Y_1_Z_1_ was (x1, y1, z1) = (0, 0, 200 mm) at coordinate system O-XYZ. Rotation angle of coordinate system O1-X_1_Y_1_Z_1_ relative to coordinate system O – XYZ was $(\theta_{x},\theta_{y},\theta_{z})$ = (-10°, 4°, t) (where t referred to the axial rotation angle of the main r intramedullary nail, with the change range from - 20° to 20°; the positive direction was external rotation, while the negative direction was internal rotation). The coordinate transformation matrix of coordinate system O_1_-X_1_Y_1_Z_1_ to coordinate system O – XYZ was defined as matrix C.

C = $\left[\begin{matrix} \text{cosθ}_{\text{y}}\text{cosθ}_{\text{z}} & -\text{cosθ}_{\text{y}}\text{sinθ}_{\text{z}} & \text{sinθ}_{\text{y}} & \text{x}_{1} \\ \text{sinθ}_{\text{x}}\text{sinθ}_{\text{y}}\text{cosθ}_{\text{z}}+\text{cosθ}_{\text{x}}\text{sinθ}_{\text{z}} & -\text{sinθ}_{\text{x}}\text{sinθ}_{\text{y}}\text{sinθ}_{\text{z}}+\text{cosθ}_{\text{x}}\text{cosθ}_{\text{z}} & -\text{sinθ}_{\text{x}}\text{cosθ}_{\text{y}} & \text{y}_{1} \\ -\text{cosθ}_{\text{x}}\text{sinθ}_{\text{y}}\text{cosθ}_{\text{z}}+\text{sinθ}_{\text{x}}\text{sinθ}_{\text{z}} & \text{cosθ}_{\text{x}}\text{sinθ}_{\text{y}}\text{sinθ}_{\text{z}}+\text{sinθ}_{\text{x}}\text{sinθ}_{\text{z}} & \text{cosθ}_{\text{x}}\text{cosθ}_{\text{y}} & \text{z}_{1} \\ 0 & 0 & 0 & 1 \end{matrix}\right]$

Note: In matrix C, θ3 = t, as the variable. The values of the transformation matrix were different due to different values of t.

According to measured data, in the coordinate system O_1_-X_1_Y_1_Z_1_, if the length of the guide pin was 100 mm and the angle between guide pin and main nail was 130°, the coordinate of the endpoint of guide pin in the coordinate system O_1_-X_1_Y_1_Z_1_ would be $(\text{-}100\times\sin(130^{\circ}),0,170)$= (76.60, 0, 170). When the main nail was in axial rotation, the coordinate of the endpoint of guide pin O_3_ would be (x_3_, y_3_, z_3_) in the coordinate system O-XYZ, thus [x_3_, y_3_, z_3_, 1]^T^= C × [-76.60, 0, 170, 1]^T^, namely coordinates of O_3_ were as follows:

$$x_{3}=\cos\theta_{y}\cos\theta_{z}\times x_{31}-\cos\theta_{y}\sin\theta_{z}\times y_{31}+\sin\theta_{y}\times z_{31}+x_{1}$$

$$y_{3}=(\sin\theta_{x}\sin\theta_{y}\cos\theta_{z}+\cos\theta_{x}\sin\theta_{z})\times x_{31}-(\sin\theta_{x}\sin\theta_{y}\sin\theta_{z}-\cos\theta_{x}\cos\theta_{z})\times y_{31}-\sin\theta_{x}\cos\theta_{y}\times z_{31}+y_{1}$$

$$z_{3}=(-\cos\theta_{x}\sin\theta_{y}\cos\theta_{z}+\sin\theta_{x}\sin\theta_{z})\times x_{31}+(\cos\theta_{x}\sin\theta_{y}\sin\theta_{z}+\sin\theta_{x}\sin\theta_{z})\times y_{31}+\cos\theta_{x}\cos\theta_{y}\times z_{31}+z_{1}$$

Where$(x_{31},y_{31},z_{31})=(-76.60,0,170)$

In the fluoroscopy coordinate system O-XYZ, coordinate of *O*_1_, *O_2_*, and *O_3_* were (0, 0, 200 mm), (7.37, 18.31, 303.87), and (x_3_, y_3_, z_3_). According to the coordinate values of *O*_1_, *O*_2_, and *O*_3_, PSA of guide pin was calculated from plane XOZ and anteversion angle of guide pin was calculated from plane YOZ.

5) To calculate the PSA, the projection of *O_1_*, *O_2_* and *O_3_* in the fluoroscopy plane at the anteroposterior position XOZ should be calculated and recorded as$O_{1}^{1},O_{2}^{1},O_{3}^{1}$. Then, the PSA was the vertex angle$\angle O_{1}^{1}O_{2}^{1}O_{3}^{1}$ of the triangle$\Delta O_{1}^{1}O_{2}^{1}O_{3}^{1}$(Figure 2E). The calculation step was:

1. The coordinate of O_1_ in the coordinate system O-XYZ was measured as$(x_{1},y_{1},z_{1})=(0,0,200)$;
2. The coordinate of O_2_ in the coordinate system O-XYZ was measured as$(x_{2}\text{，}y_{2}\text{，}z_{2})=(7.37,18.31,303.87)$;
3. The coordinate of O_3_ in the coordinate system O-XYZ was calculated and the values were different from rotation angles$\theta_{z}$:

$$x_{3}=\cos\theta_{y}\cos\theta_{z}\times x_{31}-\cos\theta_{y}\sin\theta_{z}\times y_{31}+\sin\theta_{y}\times z_{31}+x_{1}$$

$$y_{3}=(\sin\theta_{x}\sin\theta_{y}\cos\theta_{z}+\cos\theta_{x}\sin\theta_{z})\times x_{31}-(\sin\theta_{x}\sin\theta_{y}\sin\theta_{z}-\cos\theta_{x}\cos\theta_{z})\times y_{31}-\sin\theta_{x}\cos\theta_{y}\times z_{31}+y_{1}$$

$$z_{3}=(-\cos\theta_{x}\sin\theta_{y}\cos\theta_{z}+\sin\theta_{x}\sin\theta_{z})\times x_{31}+(\cos\theta_{x}\sin\theta_{y}\sin\theta_{z}+\sin\theta_{x}\sin\theta_{z})\times y_{31}+\cos\theta_{x}\cos\theta_{y}\times z_{31}+z_{1}$$

Where $(x_{31},y_{31},z_{31})=(-76.60,0,170)$,$\theta_{x}=-10^{\circ},\theta_{y}=4^{\circ}$;

1. The coordinate values of projection points$O_{1}^{1},O_{2}^{1},O_{3}^{1}$ were$(0,0,200)$,$(7.37,0,303.87)$,$(x_{3},0,z_{3})$, respectively;
2. PSA $\theta_{1}$ was obtained by triangle cosine theorem: $\cos\theta_{1}=\frac{a^{2}+b^{2}-c^{2}}{2ab}$,$\theta_{1}=\arccos(\frac{a^{2}+b^{2}-c^{2}}{2ab})$

where $a=\sqrt{(x_{1}-x_{2})^{2}+(z_{1}-z_{2})^{2}}$,$b=\sqrt{(x_{2}-x_{3})^{2}+(z_{2}-z_{3})^{2}}$,$c=\sqrt{(x_{3}-x_{1})^{2}+(z_{3}-z_{1})^{2}}$

6) To calculate the anteversion angle of guide pin, the projection of *O*_1_, *O*_2_ and *O*_3_ in the plane Y*O*Z should be calculated and recorded as $O_{1}^{2},O_{2}^{2},O_{3}^{2}$. Then, the anteversion angle of guide pin was the supplementary angle of vertex angle (Figure 2E). The calculation steps were as follow:

1. The coordinate of O_1_ in the coordinate system O-XYZ was measured as$(x_{1},y_{1},z_{1})=(0,0,200)$;
2. The coordinate of O_2_ in the coordinate system O-XYZ was measured as $(x_{2}\text{，}y_{2}\text{，}z_{2})=(7.37,18.31,303.87)$;
3. The coordinate of O3 in the coordinate system O-XYZ was calculated, and the values were different for different rotation angles : $\theta_{z}$

$$x_{3}=\cos\theta_{y}\cos\theta_{z}\times x_{31}-\cos\theta_{y}\sin\theta_{z}\times y_{31}+\sin\theta_{y}\times z_{31}+x_{1}$$

$$y_{3}=(\sin\theta_{x}\sin\theta_{y}\cos\theta_{z}+\cos\theta_{x}\sin\theta_{z})\times x_{31}-(\sin\theta_{x}\sin\theta_{y}\sin\theta_{z}-\cos\theta_{x}\cos\theta_{z})\times y_{31}-\sin\theta_{x}\cos\theta_{y}\times z_{31}+y_{1}$$

$$z_{3}=(-\cos\theta_{x}\sin\theta_{y}\cos\theta_{z}+\sin\theta_{x}\sin\theta_{z})\times x_{31}+(\cos\theta_{x}\sin\theta_{y}\sin\theta_{z}+\sin\theta_{x}\sin\theta_{z})\times y_{31}+\cos\theta_{x}\cos\theta_{y}\times z_{31}+z_{1}$$

Where $(x_{31},y_{31},z_{31})=(-76.60,0,170)$,$\theta_{x}=-10^{\circ},\theta_{y}=4^{\circ}$;

1. The coordinate values of projection points$O_{1}^{2},O_{2}^{2},O_{3}^{2}$were$(0,0,200)$, $(0,18.31,303.87)$,$(0,y_{3},z_{3})$, respectively;
2. PSA $\theta_{2}$was obtained by triangle cosine theorem:

$\cos(180^{\circ}-\theta_{2})=\frac{a^{2}+b^{2}-c^{2}}{2ab}$,$\theta_{2}=180^{\circ}-\arccos(\frac{a^{2}+b^{2}-c^{2}}{2ab})$

Where $a=\sqrt{(y_{1}-y_{2})^{2}+(z_{1}-z_{2})^{2}}$,$b=\sqrt{(y_{2}-y_{3})^{2}+(z_{2}-z_{3})^{2}}$,$c=\sqrt{(y_{3}-y_{1})^{2}+(z_{3}-z_{1})^{2}}$
